# Impact of capsaicin on aroma release: in vitro and in vivo analysis

**DOI:** 10.1016/j.foodres.2020.109197

**Published:** 2020-07

**Authors:** Ni Yang, Cassia Galves, Ana Carolina Racioni Goncalves, Jianshe Chen, Ian Fisk

**Affiliations:** a Division of Food Sciences, University of Nottingham, Sutton Bonington Campus, Loughborough LE12 5RD, UK; b Department of Food Engineering, Universidade Estadual Do Centro-Oeste – Unicentro, Paraná, Brazil; c Luiz de Queiroz College of Agriculture, University of São Paulo, Brazil; d Zhejiang Gongshang University, Hangzhou, China

**Keywords:** Chili, Spicy food, Aroma release, Saliva, APCI-MS, Breath analysis

## Abstract

Capsaicin is the main bioactive compound in chili pepper that leads to the perception of “spiciness”. However, the effect of capsaicin on aroma release in the nose remains unexplained. This is the first study designed to measure capsaicin’s impact on aroma release during consumption. In vitro studies, using static headspace analysis by atmospheric pressure chemical ionization-mass spectrometry (APCI-MS), showed no impact of capsaicin (5 ppm) on the gas-liquid partitioning equilibria of a range of aroma compounds. However, a significant reduction in aroma release was observed in vivo, during oral melting of a model ice cube system (p < 0.05) included 5 ppm capsaicin. The total release of aroma into the nasal cavity was decreased, such that only 49% of 3-methylbutanal, 60% of 1-octen-3-ol and 83% of linalool was released. This is the first evidence of capsaicin’s reduction effect on aroma release during consumption. It was also found that 5 ppm capsaicin increased saliva secretion by 75%, which may have led to the dilution of aroma compounds in the mouth and directly impacted the aroma release into the nasal cavity. The most hydrophilic compound (3-methylbutanal) was affected by capsaicin to a greater extent than the hydrophobic compound (linalool), the solvent effect of the additional saliva may explain this.

## Highlights

• The impact of capsaicin on aroma release was evaluated in a model system.
• Capsaicin did not modify equilibrium gas-liquid partitioning of aroma compounds.
• Capsaicin enhanced saliva secretion by 75%
• Capsaicin decreased in-nose aroma release of 3-methylbutanal, 1-octen-3-ol, and linalool.
• Capsaicin induced physiological changes (saliva flow) directly impacted aroma release.

## Graphical abstract

**Figure:**
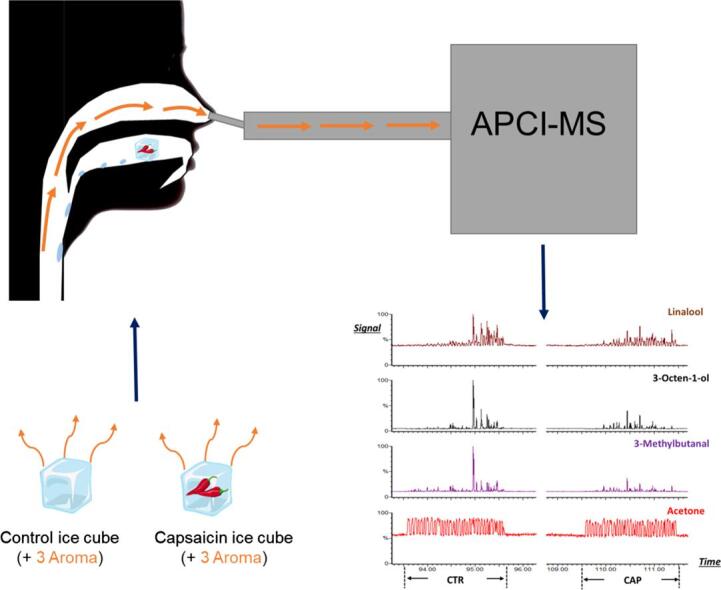

## 1 Introduction

The consumption of hot-spicy food is rapidly increasing worldwide, and many people enjoy its additional sensory contribution to food flavour. This appealing pungency is due to a group of compounds called capsaicinoids, these alkaloids, are secondary metabolites found in the plants of genus *Capsicum* (chili pepper). Naves et al. (2019) reviewed the biosynthetic pathway of capsaicinoids, and many studies have investigated the content of capsaicinoids in fresh chili (Taiti et al., 2019, Bogusz et al., 2018, Chan et al., 2018, Sganzerla et al., 2014, Ziino et al., 2009). Some models were established for the pungency prediction of capsaicinoids (Chen et al., 2019). Among all the capsaicinoids, the most abundant one is capsaicin (Cisneros-Pineda et al., 2007).

Capsaicin, an odorless and non-volatile compound, triggers the trigeminal sensation that contributes to flavour through the sensory modalities of thermal sensation and pain (Bryant & Mezine, 2002). This chemically induced sensation activates ion channels TRPV1 (transient receptor potential vanilloid subtype 1), which is known for its pain-sensitive and thermally-sensitive function. Capsaicin modulates the paracellular pathway in saliva glands and a significantly increases in saliva flow in healthy adults (Nasrawi & Pangborn, 1990; Kanehira et al., 2011). It also leads to airway sensory nerve activation and induces a secretory response of nasal fluid (Philip, Baroody, Proud, Naclerio, & Togias, 1994). Consequently, the increased level of nasal and saliva secretions could potentially influence aroma partitioning and release. Van Ruth and Buhr (2003) showed that 40% increase in saliva in a model mouth directly resulted in a 42% reduction of aroma compounds (the average of three compounds monitored). However, there is a lack of analytical evidence on how capsaicin may impact aroma release in the nasal cavity during retronasal flavour perception.

Atmospheric pressure chemical ionization-mass spectrometry (APCI-MS) is a rapid on-line analysis technique that measures the relative abundance of volatile aroma compounds in the gas phase in real-time (Taylor et al., 2000, Buffo et al., 2005, Linforth et al., 2010, Yang et al., 2011). Static headspace analysis measures the concentration above the headspace of the sample at equilibrium conditions in a closed system, this can be used to calculate the relative gas-liquid equilibrium partitioning of a compound and is typically used to evaluate the impact of changes in the aroma compounds partitioning into its carrier solvent (e.g. the impact of salt addition directly results in salting out of aroma volatile from a aqueous carrier solvent) and directly relates to a compounds availability for orthonasal flavour perception. Breath-by-breath, APCI-MS in vivo analysis can monitor real-time changes of known compounds in the nasal cavity during eating (Genovese, Yang, Linforth, Sacchi, & Fisk, 2018), this is particularly relevant for monitoring the dynamic release of aroma compounds from food materials during retronasal flavour perception. Therefore, APCI-MS offers significant advantages to standard GC-MS type approaches when determining the impact of various factors on flavour delivery. Examples include, the structure/texture of the food matrix (Tarrega, Yven, Semon, & Salles, 2008), the dynamics of oral processing (Salles et al., 2011), panelist variations (Laboure, Repoux, Courcoux, Feron, & Guichard, 2014), presence of other food ingredients such as fat (Linforth et al., 2010), proteins (Viry, Boom, Avison, Pascu, & Bodnar, 2018) and polysaccharides (Cook, Methven, Parker, & Khutoryanskiy, 2018).

Capsaicin perception and its detection limit has been shown to vary significantly between panelists, this may be due to the prior exposure and frequency of consumption (Prescott & Stevenson, 1995), personality (Byrnes and Hayes, 2013, Byrnes and Hayes, 2015), gender (Byrnes & Hayes, 2015), PROP taster status and emotions (Scott, Burgess, & Tepper, 2019). Prescott and Stevenson (1995) reported that most people rated sweetness and flavour intensity lower when capsaicin was present in a flavoured sucrose solution, but no significant impact was reported on its perceived flavour intensity when they examined the effect of capsaicin on ratings of flavour alone. So, the mechanism of how capsaicin affects flavour perception remains unclear. The impact of capsaicin on flavour perception may be due to cross-modal flavour modification as capsaicin stimulates trigeminal sensations, which interact with the perception of taste and smell (Spence, 2019). Alternatively, capsaicin may chemically interact bind with flavour compounds in the matrix, or capsaicin might change physiological conditions in the oral nasal cavity, such as saliva flow rate, which may impact aroma delivery in-nose due to the changing abundance of saliva in the oral-nasal cavity. This paper set out to evaluate the last hypothesis using APCI-MS.

The objective of this study was therefore, to investigate if capsaicin can affect aroma partitioning into the headspace of the sample (in vitro analysis) and aroma release into the nose-space during consumption (in vivo analysis). Three aroma compounds with different physicochemical properties were selected and their release was monitored by APCI-MS. Acetone, as a natural by-product of human respiration, was used to monitor exhalations in the breathing pattern.

## 2 Materials and methods

### 2.1 Chemicals

All chemicals were purchased from Sigma Aldrich and were Food Grade. Pure capsaicin is a white powder and is water-insoluble. Capsaicin (0.1 g) was pre-dissolved in food-grade ethanol (10 g) to form a 1% stock solution, this stock solution was then added into the solution at 5 ppm (mg/kg) when required. Log P and vapor pressure for the three selected aroma compounds (3-methylbutanal, 1-octen-3-ol, and linalool) were estimated by EPI Suite™ (2016) - Estimation Programs Interface Suite™ for Microsoft® Windows, v 4.1, United States Environmental Protection Agency, Washington, DC, USA.

### 2.2 Ice cube system

The matrix system was prepared in 1L pure water with the three selected aroma compounds at 100 µL each. Half of this solution (500 ml) was used as the control sample (CTR), and 250 µL of 1% capsaicin solution was added into the other half (500 ml) resulting in 5 ppm as the final concentration in the capsaicin sample (CAP). The aqueous solutions (5 ml) were pipetted into individual ice cube molds (1 mold with 18 cubes at 270 mm × 270 mm) for CTR and CAP samples, which were sealed and frozen (−18 °C) for 24 h prior to the experiment.

### 2.3 APCI-MS analysis

The MS Nose interface (Micromass, Manchester, UK) fitted to a Quattro Ultima mass spectrometer (Waters Corporation, Milford, MA) was used in this study. The selected ion mode was used with the cone voltage of 50 V, source temperature of 75 °C and a dwell time of 0.02 s. The transfer line temperature was set at 120 °C.

#### 2.3.1 In vitro analysis

For static headspace in vitro analysis, individual ice cubes were placed in 100 ml Duran bottles with a screw cap to minimise the loss of volatile compounds. These ice cubes melt completely at room temperature (20 °C) after 3 h, and three molecules in the solution were at equilibrium condition with the final solution temperature at 16–18 °C. Four replicates were prepared for each of the CTR and CAP systems and analysed in a randomized order. The headspace of each bottle was evaluated by APCI-MS when 5 ml/min of nitrogen sucked the volatiles at the headspace into the transfer line and passed to the ionization region.

#### 2.3.2 In vivo analysis

The standard technique for APCI-MS in vivo analysis was developed previously to measure in-nose aroma release (Yang et al., 2011), and the results illustrated that using four panelists with three replicates for each sample was sufficient to differentiate product difference. In this study, the same procedures were followed with four panelists recruited from the University of Nottingham (average age 23). All the panelists were regular chili consumers with at least three times a week. Every panelist was asked to cleanse their mouth with water and then took turns to place a flavoured ice cube in their mouth and breathe into the nose piece connected to the APCI-MS. They were asked to leave the ice to melt in their mouth gradually without any oral movement, in order to minimize other oral processing effects on aroma release. A portion of air (30 ml/min) was sampled from one of their nostrils, and they were asked to breathe and swallow normally for 2 min when the ice had gradually turned into water and then was swallowed completely; measurement was continued until all the target ion signals returned to the baseline. Since capsaicin can have a lasting burning sensation after consumption, each panelist was given at least a 30 min break before testing the next sample. Prior to consuming a sample, their exhaled air for all the target aroma compounds was monitored to ensure there was no carry-over aroma in their breath from the previous test. Acetone, as a natural by-product of human respiration -, was used to monitor exhalations in the breathing pattern during the in vivo study with the target ion at 59 *m*/*z*.

### 2.4 Saliva secretion measurement

Ten microliter of capsaicin solution (5 ppm) or pure water was added to a square of cotton gauze (35 mm × 35 mm × 10 mm). Each cotton gauze was weighed and then placed into the mouth. It was left on the top of the tongue with mouth closed and no oral movement for 2 min, and the weight difference at the end was regarded as the amount of saliva secreted. Four panelists were asked to cleanse their mouth with water before each test, and rest at least 30 min between samples. Each panelist’s saliva was collected for both control and capsaicin-imbedded cotton gauze three times on separate days (n = 3).

### 2.5 Ice melting time record

The time immediately after the panelists put the ice cube sample into their mouth was recorded as the starting time, and the time was also recorded when they noticed that the ice sample was melted completed. The total length of ice melting could be estimated in this way, and four panelists conducted three replicates for CAP and CTR samples respectively with a 1 h break between samples.

### 2.6 Data analysis

The maximum ion intensity (Imax) for each sample was recorded during in vitro static headspace analysis and data of four replicates (CTR samples and CAP samples) was analyzed by *t*-test for each compound to determine if capsaicin significantly affected its static release (p < 0.05).

For in vivo analysis, the area under the curve (AUC), time to reach the maximum intensity (Tmax) and Imax were extracted from the chromatogram of the APCI-MS. Multivariate analysis of variance (IBM® SPSS® Statistics version 21.0.0) was applied to evaluate for significant differences (p < 0.05) between the CTR and the CAP samples, where human subjects used as a fixed effect in the analysis.

## 3 Results

### 3.1 Method and system validation

More than 300 individual volatiles have been found in chili pepper (Rodríguez-Burruezo, Kollmannsberger, González-Mas, Nitz, & Fernando, 2010), and those aroma compounds can be classified into different functional groups with different physicochemical properties. However, it is reasonable to select a few compounds for APCI-MS real-time analysis to optimize data capture speed and analytical sensitivity. Therefore, three common aroma compounds that are found in chili, which have different physicochemical properties were selected (Table 1). According to the hydrophobicity (Log P) and vapour pressure (VP), capsaicin is the most hydrophobic and the least volatile compound. Among the selected aroma compounds, the most hydrophilic and the most volatile compound was 3-methylbutanal, linalool was the most hydrophobic and least volatile compound, whilst 1-octen-3-ol was intermediate.

**Table 1:** The physicochemical properties of three selected aroma compounds, and capsaicin.

Compounds	Molecular Mass (Da)	Target Ion (*m*/*z*)	Signal variation (%CV#, n = 4)	Hydrophobicity (Log P##)	Volatility (VP##, 25 °C, mm Hg)	Air-water partitioning coefficient (Kaw##)
3-Methylbutanal	86	87	3%	1.23	51.60	6.49E−03
1-Octen-3-ol	128	111	4%	2.73	0.24	9.45E−04
Linalool	154	137	7%	3.38	0.08	1.73E−03
Capsaicin	305	NA	NA	4.0	1.32E −08	4.23E −12

# CV% = 100 × Standard Deviation/Mean.

## The value of Log P, vapour pressure (VP) and Kaw were estimated by EPI Suite™ v.4.1 software, U.S. Environmental Protection Agency.

The APCI signal depends on the fragments of the compound after soft ionization with the addition of a proton (H+). The target ion for 3-methylbutanal was 87 based on its molecular mass 86 plus 1 for the proton. Whilst, 1-octen-3-ol (Mw = 128) and linalool (Mw = 154) have a hydroxyl group (–OH) that can form water molecule with H+, so their target ions were their respective molecular mass plus 1 and minus the molecular weight of water (18), giving the respective target ion as 111 and 137 (Table 1). The signal for the individual compounds at their respective target ions was reproducible (CV% = 3–7%) from four replicated standard solutions by APCI-MS static headspace analysis. Therefore, it was concluded that the method was valid to study the effect of capsaicin on aroma release in-vitro using static headspace by APCI-MS.

**Fig. 1:**
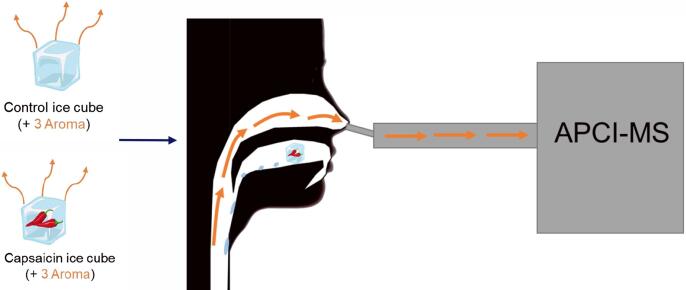
Schematic diagram of experimental design for the *in-vivo* study.

A simple food matrix was required to demonstrate the effect of capsaicin on aroma release whilst minimizing variations in oral processing. The simplest system, an aqueous solution, has been shown to be sensitive to intra- and inter-panelist variations (Linforth and Taylor, 2000, Weel et al., 2003). This variation is due to the fact that aroma release is largely dependent on a single time point after swallowing (1–2 s). To overcome this, a flavoured ice cube system was used in this study, which could melt inside the mouth with the minimum of oral movement; whole aroma release via the retronasal route could be monitored during a prolonged consumption period (1–2 min). Using the ice cube system not only helped to generate reproducible data with a steady aroma release but also created a cooling sensation that might mitigate the pain and heat sensation generated by capsaicin. Fig. 1 shows a schematic diagram of the experimental design for in-vivo study.

The level of the capsaicin used in this study is 5 ppm, which is equivalent to 75 SHU (Scoville Heat Units), calculated by multiplying ppm by a factor of 15 (Collins, Wasmund, & Bosland, 1995). Green peppers have reported as 1.0 ± 0.9 ppm and regarded as non-pungent (Othman, Ahmed, Habila, & Ghafar, 2011). In this study, 5 ppm was used as it is in the middle of the range used in previous capsaicin oral exposure studies (Lv et al., 2019, Prescott and Stevenson, 1995). This level was found to give a sense of hotness without causing pain in the mouth.

**Fig. 2:**
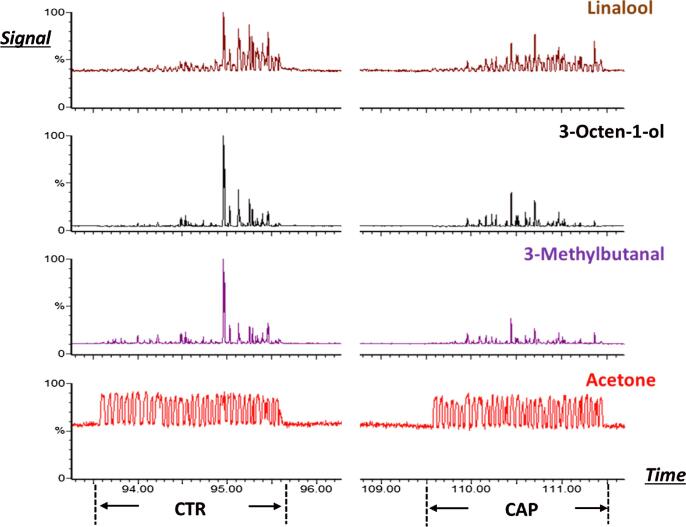
Breath-by-breath signals for four compounds (linalool, 3-octen-1-ol, 3-methylbutanal and acetone) during consumption measured by APCI-MS for a control sample (CTR) at time 93.5–95.5 min and a capsaicin sample (CAP) at time 109.5 – 111.5 min.

When a panelist consumed an ice cube containing either 5 ppm capsaicin or a blank, the release of aroma compounds into the nasal cavity was monitored in real-time using APCI-MS (Fig. 2). Acetone is a marker for an exhaled breath, and the acetone peaks can be used to illustrate the pattern of breathing. The release patterns of all three target compounds during consumption of the capsaicin samples (CAP) and the control samples (CTR) were clearly demonstrated in this chromatogram, from which parameters detailing aroma release (Imax, AUC, and Tmax) were extracted. Therefore, APCI-MS in-nose release using the ice cube system was proved as a suitable method to show the impact of capsaicin on aroma release during consumption.

### 3.2 Impact of capsaicin on in vitro aroma release

During in vitro static headspace analysis, aroma compounds partition between air and aqueous solutions at equilibrium in a sealed container, the equation for air-water partitioning coefficient (Kaw) is $\mathrm{Kaw}=\frac{\mathrm{Cair}}{\mathrm{Cwater}}$, which can be rearranged to show the concentration of the aroma compound above headspace of the samples is Cair=Kaw×Cwater. All three compounds were added at the same level (100 ppm) into the solution, so the higher the Kaw value, the higher the Cair. It was assumed that 3-methyl butanal with the largest Kaw (Table 1) had the highest Cair. However, 1-octen-3-ol showed the highest ion intensity (Table 2) because APCI signal also depend on other factors that affect how many target ions reach the detector, such as volatility, fragmentation, and interactions with the tubing materials. Fragile aldehyde compounds (sensitive to oxygen, temperature and moisture) like 3-methylbutanal might be degraded after passing through the hot transfer line and so fewer molecules are left to reach the detector.

**Table 2:** The ion intensity of each aroma compound at equilibrium measured by headspace analysis for the control samples (CTR with 4 replicates a, b, c and d) and capsaicin samples (CTR with 4 replicates – a, b, c and d) and its respective average value (AV), standard deviation (SD) and CAP/CTR Ratio.

	3-Methylbutanal	1-Octen-3-ol	Linalool
CTR a	8.47E+07	9.65E+08	2.70E+07
CTR b	9.19E+07	1.05E+09	3.18E+07
CTR c	8.78E+07	9.98E+08	2.90E+07
CTR d	8.86E+07	9.89E+08	2.95E+07
***AV (CTR)***	*8.83E*+*07*	*1.00E*+*09*	*2.93E*+*07*
***SD (CTR)***	*2.96E*+*06*	*3.58E*+*07*	*1.97E*+*06*
CAP a	9.32E+07	1.05E+09	3.47E+07
CAP b	8.98E+07	9.98E+08	2.80E+07
CAP c	9.48E+07	1.07E+09	3.46E+07
CAP d	9.20E+07	9.93E+08	3.16E+07
***AV (CAP)***	*9.25E*+*07*	*1.03E*+*09*	*3.22E*+*07*
***SD (CAP)***	*2.11E*+*06*	*3.82E*+*07*	*3.16E*+*06*
**CAP/CTR Ratio**#	**1.0**	**1.0**	**1.1**

# CAP/CTR ratio was calculated by AV (CAP) divided by AV (CTR), if the ratio equals 1, the maximum release from capsaicin samples is similar to its maximum release from the control samples.

Nevertheless, the ion intensity of the four replicated CTR samples and the four replicated CAP samples are shown in Table 2, and its release ratio (CAP / CTR) was calculated for each compound. The ratio for all aroma compounds was close to 1, so the release between the two systems was similar, and statistical tests showed no significant difference between capsaicin and the control samples for each compound (p > 0.05). Capsaicin is a very hydrophobic compound that might chemically interact with other aroma compounds or physically bind with other ingredients, such as starch (Schneider, Seuß-Baum, & Schlich, 2014) and fat (Nolden, Lenart, & Hayes, 2019). Our headspace results indicate that no measurable chemical or physical interactions occurred between the aroma compounds and capsaicin in the ice cube system. However, this might not apply in a more complicated food system where capsaicin and aroma compounds could be physically trapped by fat or chemically bound by protein or other reactive species.

In this ice cube system, capsaicin had no impact on aroma release in vitro whilst under static equilibrium headspace conditions, so any aroma difference observed during oral consumption of the capsaicin samples is more likely due to the effect of capsaicin on the physical or physiological changes inside mouth and nose.

### 3.3 Impact of capsaicin on in vivo release during consumption

**Fig. 3:**
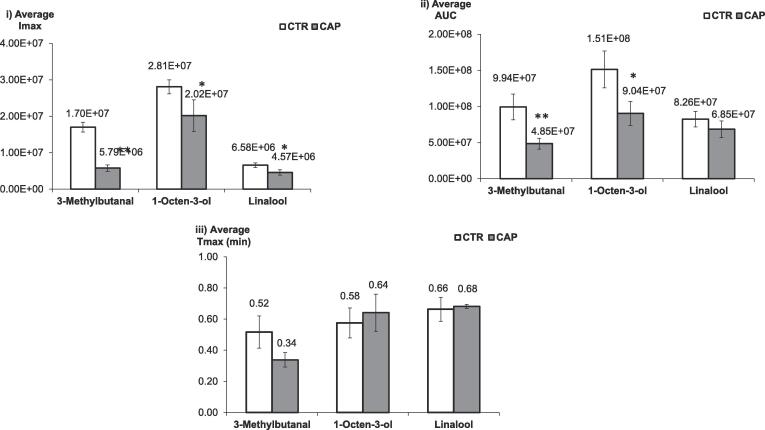
The average (i) Imax and (ii) AUC, (iii) Tmax results of each aroma compound released in-nose during consumption of control (CTR, white bar) and capsaicin (CAP, grey bar) samples. Standard error is shown as +/- error bars, * and ** indicated a statistical difference of p < 0.05 and p < 0.01 as measured by ANOVA.

The average signal (Imax, AUC, and Tmax) of each compound was calculated for CTR and CAP samples (Fig. 3). Statistically, there is a significant difference between CTR and CAP on the average Imax of 3-methylbutanal (p < 0.01), 1-octen-3-ol (p < 0.05) and linalool (p < 0.05). Capsaicin caused a significant reduction on the total in-nose release (AUC) of 3-methylbutanal (p < 0.05) and 1-octen-3-ol (p < 0.05). Although the time for each compound to reach its maximum (Tmax) was not significantly different, the results of Imax and AUC in this study provide the first analytical evidence to demonstrate the significant impact of capsaicin on aroma release in vivo.

**Fig. 4:**
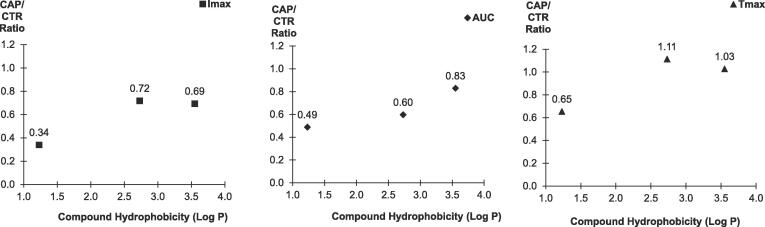
Compound hydrophobicity (Log P) and the ratio of CAP/CTR for average Imax (■), AUC (♦), and Tmax (▲) values from capsaicin and control samples.

When comparing the aroma release difference between two products, the data can be better compared by calculating the ratio of its release, so some of the “noise” in the raw data is removed and the aroma release difference between two systems is clearer (Shojaei, Linforth, Hort, Hollowood, & Taylor, 2006). In this study, the release ratio (CAP / CTR) for each aroma compound was calculated using its average value from CAP samples divided by its average from CTR samples. The hydrophobicity of the target compounds was plotted against this ratio for average Imax, AUC, and Tmax (Fig. 4). It is shown that the Imax for all three compounds had CAP/CTR ratios of less than 1.0, which indicated that the maximum level of aroma release was lower in CAP than CTR samples. The most hydrophilic compound 3-methylbutanal (Log P = 1.23) had an CAP Imax value of 34% of CTR (100%), whilst the other two compounds had an CAP Imax value of around 69–72% of CTR (100%).

Similarly, the CAP/CTR ratios for AUC for all three compounds are lower than 1.0, so the total amount of aroma released in the nose is much lower in the CAP samples. Only 49% of AUC was observed for the most hydrophilic compound when comparing CAP to CTR, so capsaicin caused a 51% reduction of its total release in-nose. The most hydrophobic compound had the least reduction of up to 17%.

Regarding the ratio of CAP and CTR for Tmax (Fig. 4), the most hydrophilic compound had a ratio of 0.65, which might indicate that average time to reach to its maximum release is 35% quicker in capsaicin samples. However, the hydrophobic ones had a ratio near to 1.0, overall no significant impact was observed.

**Fig. 5:**
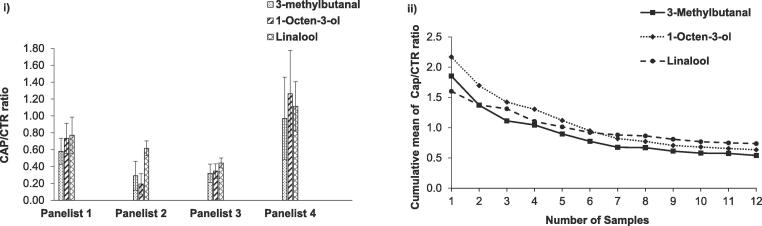
(i) Ratio of in-nose release (CAP/CTR), as measure by APCI-MS, calculated from average Imax of an individual panelist’s data for three aroma compounds (3-methylbutanal, 1-octen-3-ol and linalool). Standard error is shown as +/- error bars. (ii) Cumulative mean calculations of the CAP/CTR ratio from the Imax data for three aroma compounds (3-methylbutanal, 1-octen-3-ol and linalool). Each line represents a cumulative mean calculation for 12 samples.

In addition, intra-/inter- panelist’s variations on in vivo aroma release were observed in this study (Fig. 5i) and also in other studies (Gierczynski et al., 2008, Ruijschop et al., 2009). Comparing the CAP/CTR ratios of Imax for three target compounds between individual panelists, Panelist 1, 2 and 3 had relatively small ratios with smaller error bars compared to Panelist 4 with larger ratios and error bars. Hence, it is necessary to evaluate if a total of 12 samples (4 panelists with 3 replicates) are sufficient to represent the larger group. The “cumulative mean” approach was used by Yang et al. (2011) to illustrate the minimum number required in their aroma release in vivo study. A similar approach was applied: the CAP/CTR ratio of Imax data for an individual sample was added one at a time with the mean taken as the value for each sample was added (data were shown in the Appendix A). Fig. 5ii) demonstrated that the starting points for all three compounds varied for the first four samples, but their levels stayed relatively fixed after the values obtained from the fifth samples. Generally, the cumulative mean of the ratios for the most hydrophilic compound (3-methylbutanal) was higher than that of 1-octen-3-ol and linalool. This demonstrated that the use of these 12 samples with their CAP/CTR ratios in this study should provide sufficient data to illustrate the effect of capsaicin on in vivo aroma release during consumption.

### 3.4 Impact of capsaicin on breathing, saliva secretion and ice melting time

There is an assumption that the oral stimulation from capsaicin might change a person’s breathing pattern, we maybe more likely to hold our breath so less aroma can be pushed into the nose. The level of acetone from the breath was used as an indication of the breathing pattern, and its average AUC results were calculated for CAP samples and CTR samples. The acetone present in the breath flow during consumption of the CAP samples (1.74E6 ± 1.18E5) is lower than its level from the CTR samples (1.89E6 ± 5.11E4) and there is a significant difference between them (p < 0.05). Around 92% acetone monitored during 2 min consumption of CAP samples was compared to its release from CTR (100%). This means that the presence of capsaicin caused the panelists to suppress breathing by 8%. However, this effect is not as strong as its impact on aroma in-nose release observed previously.

Another theory is that the trigeminal stimulation of capsaicin can alter the secretion of saliva and nasal mucus, and more hydrophilic compounds are more likely to be retained in the additional saliva and nasal mucus, so less aroma is released. Therefore, the saliva secretion in the mouth was evaluated and the results are shown in Table 3. Saliva collected from control gauze was 1.88 g, and this was significantly increased when 5 ppm of capsaicin was added to the gauze (P < 0.01), with a total of 3.30 g saliva being generated. This additional 74% saliva was produced as a response to capsaicin, which could be the main reason for reduced aroma release in the nose. Nasrawi and Pangborn, 1990, Kanehira et al., 2011 also reported that saliva secretion more than doubled when stimulated by capsaicin. Additionally, other studies performed with in-mouth simulators also showed a significant decrease in aroma compound release when saliva volume increased (Mehinagic et al., 2004, Odake et al., 1998, van Ruth and Roozen, 2000). Enhanced saliva production is likely to dilute aroma compounds in the mouth and reduce their bioavailability into the nose, and this effect is often more apparent for the more hydrophilic compounds. Therefore, the physicochemical properties of the aroma compounds like hydrophobicity can play a significant role in the extent of the impact of capsaicin on aroma release.

**Table 3:** The amount of saliva (g) collected from the blank control cotton (CTR) or capsaicin-containing cotton (CAP). Ice melting time in the mouth (s) comparing CTR and CAP samples with respective average value (AV), standard deviation (SD) and CAP/CTR Ratio.

	Saliva collected (g)	Ice melting time in mouth (s)
***CTR (AV)***	***1.88***	***148***
*CTR (SD)*	*0.45*	*11*
***CAP (AV)***	***3.30***	***162***
*CAP(SD)*	*0.59*	*14*
**CAP/CTR Ratio**	**1.75**	**1.09**

Additional experiment was conducted in-vitro to evaluate the hypothesis that the observed aroma release decrease was due to the dilution of additional saliva. The control solution was 5 ml flavoured solution (sample C, n = 3), and 1.42 g saliva was added to sample C (sample S, n = 3). The headspace results showed that additional saliva had similar dilution effect to all three compounds (S/C = 0.87), which were dissimilar to previously capsaicin impact results where more hydrophilic compound (3-methyl butanal) had a larger impact than more hydrophobic ones (1-octen-3-ol and linalool). Therefore, the impact of capsaicin on aroma release might not just a simple dilution effect. However, comparing with the simple solution used in this in vitro test, the ice-cube system was used in vivo test and it melted in the mouth along with secreted saliva during the consumption period of 2 min, so in vitro aqueous situation might not fully mimic this in vivo situation. The impact of capsaicin on aroma release in vivo could involve a more dynamic and complex oral process, the dissolved volatiles were also swallowed along with the swallowing of saliva. The hydrophilic molecules that dissolved better in the saliva could be reduced at a larger extent than hydrophobic ones, which could lead to the reduction of their release signals.

Moreover, it was unknown if there could be any impact of capsaicin on ice metling rate using the ice cube during in vivo consumption. Lv et al. (2019) observed a significant temperature increase of 1.34 °C at the tip of the tongue when exposed to 20 ppm capsaicin solution. It was also reported that a 5 ppm capsaicin solution resulted in an increase in the temperature on the tongue-tip of 0.36 °C (29.87 °C compared with 29.51 °C for the control, P < 0.05). In order to evaluate if this 0.36 °C increase in tongue temperature would affect the ice cube melting time in the mouth, extra experiments were conducted and the average ice melting time of CTR and CAP ice samples was calculated in Table 3. The slight increase in tongue temperature did not speed up the ice melting rate, in fact that the opposite was true, the CAP ice samples melted about 14 s more slowly than the CTR ice samples (p < 0.05). The main reason proposed is that ice is more likely to be protected from melting with the additional saliva stimulated by capsaicin. This finding could lead to an exciting application in ice lolly or ice cream that might melt more slowly in the mouth if a spicy flavour is added.

## 4 Discussion

The hypothesis proposed in this study is that the in-nose release of more hydrophilic compounds are likely to be more affected by capsaicin than hydrophobic compounds, and the underlying reason is due to the additional saliva stimulated by capsaicin. Three target compounds with different hydrophobicity were chosen in this study and clearly illustrate this trend. However, it is recommended to use more aroma compounds to test the hypothesis in a future study. Ayed et al. (2014) conducted a static headspace analysis of 51 aroma molecules released from simple gels models, and they noticed that the Family factor, which refers to compounds with different functional groups (alcohols, aldehydes, ketones, and esters), is more significant than the Matrix factor and the Structure factor. It would be interesting to investigate if aroma release behaviour for compounds from different functional groups with different hydrophobicity would be impacted by capsaicin in a similar way.

Aroma release can also be affected by saliva via interactions between aroma compounds and constituents of saliva such as mucin and alpha-amlylase (Friel and Taylor, 2001, Dinu et al., 2019). Pages-Helary, Andriot, Guichard, and Canon (2014) also found that esters are more affected by saliva than ketone due to the presence of esterase activity in saliva. It would be interesting in future studies to explore whether capsaicin alters the composition and concentration of proteins in saliva and whether capsaicin interacts with other salivary components.

Furthermore, capsaicin has been reported to influence the sensitivity of the taste of salt in human subjects (Narukawa, Sasaki, & Watanabe, 2011), and enjoyment of spicy food was found to reduce individual salt preference with increased activity of the orbitofrontal cortex, resulting in reduced daily salt intake and blood pressure (Li et al., 2017). Other studies proposed that activation of capsaicin receptor TRPV1 had a depressor effect on an endovanilloid (N-arachidonoyl-dopamine) that is associated with a blood pressure response for high sodium intake (Wang and Wang, 2007, Hao et al., 2011, Yu and Wang, 2011). Based on the findings in our study, we could propose another mechanism, that is, the extra saliva stimulated by capsaicin increases the hydration rate of the food matrix, so sodium can be more quickly released from the matrix and delivered to the tongue, resulting in enhanced saltiness perception. Therefore, adding capsaicin or chili might help reduce the level of salt added to food products.

This study used a simple ice cube system with minimal oral processing allowed, but our chewing activity and frequency of velum opening could be affected when capsaicin-containing food is consumed. Similar analytical methods could be applied to food with more complex structures and texture, so the impact of capsaicin on oral processing could be assessed. According to Laboure et al. (2014), oral physiology of the subjects and their food oral processing could affect the total amount of aroma release during cheese consumption. Therefore, when food with a more complex texture is involved, a larger number of panelists would need to be recruited because higher inter-individual variability on aroma release in the nose during consumption is likely to be observed.

The individual difference between subjects was also observed by Byrnes, Loss, and Hayes (2015), who reported that individual food involvement and culinary experience could result in a diverse sensory perception. So a further study could involve panelists with a range of prior chili consumption frequency, and the effect of capsaicin aroma release could be linked to perceived sensory traits, which will further reveal if any aroma perception difference is associated with an in-nose release difference or whether there is a cross-modal interaction.

## 5 Conclusion

This study evaluated the impact of capsaicin on in vivo aroma release for the first time. Although no significant impact of capsaicin was observed on the static in vitro aroma partitioning, the presence of capsaicin at 5 ppm in the mouth significantly reduced the delivery of volatile aroma compounds to the nasal cavity. The hydrophilic compound 3-methylbutanal reduced by 51%, this was much greater than the more hydrophobic compound linalool which only reduced by 17%. Saliva production increased by 75% as a response to capsaicin in the oral cavity, which supports our hypothesis: that capsaicin increases saliva production, that enhanced saliva/mucus production leads to the active dilution of aroma compounds in the oral-nasal cavity during consumption and dissolved compounds was sawllowed along with the swallowing of saliva, so the level of aroma compounds being released was reduced, which is more evident for more hydrophilic compounds.

The developed model ice system proved to be successful with good release signals measured during consumption. However, this model system might have limited application, so it is worthy applying the developed analytical methods to other food matrices. Another limitation is that only three representative aroma compounds were selected and used to demonstrate the proposed mechanisms, but more aroma compounds with different hydrophobicity could be used in a future study to validate this theory. These three aroma compounds were added at analytical levels (100 ppm) in order to achieve good signals during breath-by-breath analysis, but the resulted flavour might not be considered as a typical food flavour, which made it challenging to compare with sensory perception. Further studies could link the aroma release with its perception by using a simple aroma compound or a mixture of compounds that creates a particular savory flavouring for food applications.

Nevertheless, these novel findings may aid food producers in both new and existing product development activities, especially when modifying flavour profiles to develop new healthy product ranges of food materials (e.g. low sodium seasoning variants).

## 6 Ethical review

Ethics approval (SBREC170129A) was obtained from the School of Biosciences at the University of Nottingham (July 9th 2018), and all test procedures followed the ethical rules and regulations set by the University.

## CRediT authorship contribution statement

**Ni Yang:** Conceptualization, Methodology, Formal analysis, Investigation, Validation, Writing - original draft, Writing - review & editing, Resources, Funding acquisition, Project administration, Supervision. **Cassia Galves Souza:** Formal analysis, Methodology, Investigation, Validation. **Ana Carolina Racioni Goncalves:** Formal analysis, Methodology, Investigation, Validation. **Jianshe Chen:** Funding acquisition, Writing - review & editing. **Ian Fisk:** Supervision, Writing - review & editing.
