# Supplementary material for: Impact of capsaicin on aroma release: in vitro and in vivo analysis
Source: Food Res Int. 2020 Jul;133:109197. doi: 10.1016/j.foodres.2020.109197 (PMC7262593; doi:10.1016/j.foodres.2020.109197)
Supplement: Supplementary data 1 [file mmc1.docx]

**Appendix**

Table A.1. The CAP/CTR ratios of the Imax data for an individual sample from 1 to 12, and the cumulative mean CAP/CTR for 3-methylbutanal, 1-octen-3-ol, and linalool.

| Sample | Cumulative sample | 3-Methylbutanal | | 1-Octen-3-ol | | Linalool | |
| --- | --- | --- | --- | --- | --- | --- | --- |
|  |  | CAP/CTR ratio | Cul mean CAP/CTR | CAP/CTR ratio | Cul mean CAP/CTR | CAP/CTR ratio | Cul mean CAP/CTR |
| 1 | 1 | 1.85 | 1.85 | 2.17 | 2.17 | 1.60 | 1.60 |
| 2 | 12 | 0.89 | 1.37 | 1.22 | 1.70 | 1.15 | 1.37 |
| 3 | 123 | 0.59 | 1.11 | 0.88 | 1.42 | 1.19 | 1.31 |
| 4 | 1234 | 0.84 | 1.04 | 0.95 | 1.30 | 0.47 | 1.10 |
| 5 | 12345 | 0.31 | 0.90 | 0.38 | 1.12 | 0.65 | 1.01 |
| 6 | 123456 | 0.16 | 0.77 | 0.09 | 0.95 | 0.45 | 0.92 |
| 7 | 1234567 | 0.08 | 0.68 | 0.06 | 0.82 | 0.64 | 0.88 |
| 8 | 12345678 | 0.63 | 0.67 | 0.43 | 0.77 | 0.75 | 0.86 |
| 9 | 123456789 | 0.15 | 0.61 | 0.18 | 0.71 | 0.37 | 0.81 |
| 10 | 12345678910 | 0.28 | 0.58 | 0.45 | 0.68 | 0.40 | 0.77 |
| 11 | 1234567891011 | 0.53 | 0.57 | 0.42 | 0.66 | 0.56 | 0.75 |
| 12 | 123456789101112 | 0.17 | 0.54 | 0.40 | 0.63 | 0.59 | 0.74 |
